# Methanol-Independent Protein Expression by *AOX1* Promoter with *trans*-Acting Elements Engineering and Glucose-Glycerol-Shift Induction in *Pichia pastoris*

**DOI:** 10.1038/srep41850

**Published:** 2017-02-02

**Authors:** Jinjia Wang, Xiaolong Wang, Lei Shi, Fei Qi, Ping Zhang, Yuanxing Zhang, Xiangshan Zhou, Zhiwei Song, Menghao Cai

**Affiliations:** 1 State Key Laboratory of Bioreactor Engineering, East China University of Science and Technology, 130 Meilong Road, Shanghai 200237, China; 2 Shanghai Collaborative Innovation Center for Biomanufacturing, 130 Meilong Road, Shanghai 200237, China; 3 Bioprocessing Technology Institute, 20 Biopolis Way, #06-01 Centros, Singapore; 4 Department of Biochemistry, Yong Loo Lin School of Medicine, National University of Singapore, 21 Lower Kent Ridge Road, Singapore

## Abstract

The *alcohol oxidase 1* promoter (P_*AOX1*_) of *Pichia pastoris* is commonly used for high level expression of recombinant proteins. While the safety risk of methanol and tough process control for methanol induction usually cause problems especially in large-scale fermentation. By testing the functions of t*rans*-*acting* elements of P_*AOX1*_ and combinatorially engineering of them, we successfully constructed a methanol-free P_*AOX1*_ start-up strain, in which, three transcription repressors were identified and deleted and, one transcription activator were overexpressed. The strain expressed 77% GFP levels in glycerol compared to the wide-type in methanol. Then, insulin precursor (IP) was expressed, taking which as a model, we developed a novel glucose-glycerol-shift induced P_*AOX1*_ start-up for this methanol-free strain. A batch phase with glucose of 40 g/L followed by controlling residual glucose not lower than 20 g/L was compatible for supporting cell growth and suppressing P_*AOX1*_. Then, glycerol induction was started after glucose used up. Accordingly, an optimal bioprocess was further determined, generating a high IP production of 2.46 g/L in a 5-L bioreactor with dramatical decrease of oxygen consumption and heat evolution comparing with the wild-type in methanol. This mutant and bioprocess represent a safe and efficient alternative to the traditional glycerol-repressed/methanol-induced P_*AOX1*_ system.

The methylotrophic yeast *Pichia pastoris* is a widely used expression system beyond *Escherichia coli* for production of heterologous proteins^1^,^2^. The popularity of this expression system mainly attributes to its ability of strongly and strictly controlled methanol-induced *alcohol oxidase 1* promoter (P_*AOX1*_) to produce foreign proteins at high levels^3^. This heterologous protein expressing system has several advantages over other eukaryotic and prokaryotic expression systems: high levels of foreign proteins can be expressed under the methanol-induced P_*AOX1*_; ability to grow high cell-density with rapid growth rate and almost protein-free medium; elimination of negative effects of endotoxin and bacteriophage; diverse posttranslational modifications like polypeptide folding, glycosylation, methylation, acylation, proteolytic adjustment, and targeting to subcellular compartments^1^,^4^,^5^. However, as we know, methanol is potentially hazardous for its toxicity and flammability in the large-scale fermentation industry and, methanol catabolism requires huge oxygen coupled with considerable heat release^6^,^7^. The need for high oxygen supply and heat removal increases the production cost and causes difficulties in scale-up when heat exchange and oxygen transfer capacities are low; and also, the cost of methanol will certainly increase once the oil crisis comes^5^,^8^,^9^,^10^,^11^,^12^,^13^.

In order to avoid the indispensable of methanol in *P. pastoris*, the use of alternative promoters, such as P_*GAP*_, P_*ICL1*_ and P_*PHO89*_, have been proposed^14^,^15^,^16^. However, these promoters, even after improvement^17^, have not been as widely used as P_*AOX1*_, mainly because of their weak expression ability or uncontrolled behavior. Rewiring the P_*AOX1*_ has led to various activations of P_*AOX1*_^18^,^19^, while it seemed not well eliminating the depressions caused by alternative carbon sources like high levels of glucose and glycerol. Although *cis*-acting approach on rewiring P_*AOX1*_ has been reported by several groups, *trans*-acting approach has not been well studied currently. As reported currently, several *trans*-acting elements have been reported to regulate methanol induction, glucose or glycerol derepression in *P. pastoris*. Three transcription factors, Mit1, Mxr1, and Prm1, which positively regulated P_*AOX1*_ in response to methanol^20^,^21^,^22^, were proved to bind to P_*AOX1*_ at different sites and did not interact with each other^20^. They activated P_*AOX1*_ through a cascade^20^. Besides, deletion of either the hexose transporter Hxt1^23^ or the glucose sensor Gss1^24^ slightly derepressed P_*AOX1*_ in response to glucose. Nrg1 suppressed P_*AOX1*_ in glucose and glycerol by directly binding to five sites of P_*AOX1*_, including two binding sites of transcriptional activator Mxr1^25^.

Although many efforts have been made to facilitate methanol-free heterologous protein expression driven by P_*AOX1*_ in *P. pastoris*, there is still a long way to go, especially for the industrial use. In this work, we are committed to engineer a new methanol-free *P. pastoris* by modifying transcription factors of *AOX1* promoter and developing its accessional bioprocess control for protein expression. Accordingly, an efficient glucose-glycerol-shift induction in the newly constructed strain was established to replace the traditional glycerol-methanol induction in the wild-type for protein expression. It exhibited an economic, safe and environment-friendly pattern comparing with the traditional methanol-dependent expression system, and so that holds great potential for industrial application.

## Results and Discussion

### Characterization of transcriptional repressors and their combinatorial functions on *AOX1* promoter in *P. pastoris*

**Figure 1:**
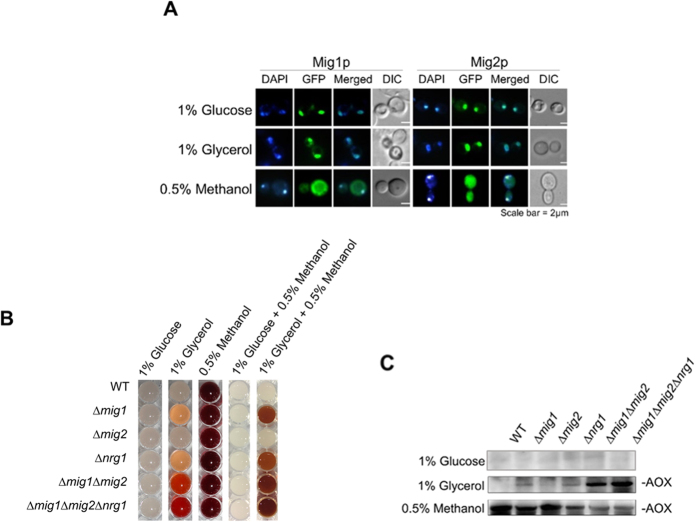
Deficiency of transcription repressors conditionally derepressed AOX expression by P_*AOX1*_. (**A**) Subcellular localization of GFP-Migl or GFP-Mig2 under repressing (glucose or glycerol) or inducing (methanol) conditions. Wild-type (WT) strains expressing GFP-Migl or GFP-Migl were grown on YNB + 0.5% Methanol, YNB + 1% glucose or YNB + 1% glycerol. The cells were stained with DAPI and then imaged for GFP and DAPI fluorescence and by Nomarski optics. Scale bar = 2 μm. (**B**) Colorimetric AOX enzyme assays of WT, Δ*mig1*, Δ*mig2*, Δ*nrg1*, Δ*mig1*Δ*mig2* and Δ*mig1*Δ*mig2*Δ*nrg1* strains in different carbon sources. Cells were grown in YND (YNB + 1% glucose) medium for 2 days and then washed three times by sterile water and transferred into 1% glucose, 1% glycerol, 0.5% methanol, 1% glucose + 0.5% methanol or 1% glycerol + 0.5% methanol-containing medium. Four hours later, AOX activity was visualized by adding the AOX activity reaction mixture. (**C**) Western blot detection of AOX protein in WT, Δ*mig1*, Δ*mig2*, Δ*nrg1*, Δ*mig1*Δ*mig2* and Δ*mig1*Δ*mig2*Δ*nrg1* strains induced on different carbon sources. Original gels/blots are provided in Supplementary Figure S2.

In *S. cerevisiae*, ScMig1, ScMig2 and ScMig3 are known to repress the transcription of genes involved in other carbon source metabolism in the presence of glucose^26^,^27^. A BLAST search of the *P. pastoris* genome^28^ identified two homologues of ScMig: Mig1 (GenBank accession number CAY71576), Mig2 (GenBank accession number CAY68378). Mig1 and Mig2 consist of 445 and 455 amino acids, respectively, and both belong to the family of Cys_2_His_2_ proteins, which located close to the N-terminus (Supplementary Fig. S1). In *S. cerevisiae*, glucose promotes nuclear localization and thus the activity of ScMig1^29^. A similar mechanism may happen in *P. pastoris*. Using live cell imaging of GFP tagged Mig1 or Mig2, we found that Mig1 and Mig2 were localized in the nucleus in cells grown in glucose or glycerol, as expected for glucose- or glycerol- dependent transcriptional repressors. When *P. pastoris* cells were transferred to methanol, Mig1 and Mig2 were predominantly localized in the cytoplasm (Fig. 1A). These data suggested that Mig1 and Mig2 might be critical for catabolite repression of the P_*AOX1*_.

In order to elucidate their roles in the regulation of P_*AOX1*_, *MIG1* and *MIG2* were knocked out independently. Besides, we have identified another important transcription repressor Nrg1 of P_*AOX1*_^25^. Since Mig1, Mig2 and Nrg1 may have redundant functions in transcriptional regulation, a double deletion Δ*mig1*Δ*mig2* mutant and a triple deletion Δ*mig1*Δ*mig2*Δ*nrg1* mutant were also generated. A colorimetric assay^23^ was used to reflect the alcohol oxidase (AOX) activity (driven by native P_*AOX1*_). Accordingly, high levels of AOX were detected in wild-type and the mutants in response to 0.5% methanol, no AOX activity was detected in any of the cells in the presence of 1% glucose (Fig. 1B). The expression of AOX in wild-type and Δ*mig2* cells was suppressed in 1% glycerol. However, all other mutants expressed certain amount of AOX in response to 1% glycerol and the activity of AOX was high in the double and triple mutants. Moreover, for all strains, 1% glucose strictly repressed AOX expression even coexisted with 0.5% methanol, meaning that methanol could not induce AOX expression under glucose condition. These results were different from those in *Hansenula polymorpha* (methanol induced AOX expression even with 1% glucose in Δ*mig1* and Δ*mig1*Δ*mig2* mutants)^30^ but similar to those in *Candida boidinii* (glucose repressed activity of the *AOD1* promoter even with methanol existed)^31^. However, mixed carbon source of 1% glycerol +0.5% methanol generated similar derepression effects of AOX expression to 1% glycerol but with higher expression levels which may ascribe to methanol induction (Fig. 1B). Western blot results showed a clear correlation between the colorimetric reactions and the amount of AOX protein in different cells (Fig. 1C). These results showed that AOX expression in wild-type cells was tightly suppressed by 1% glycerol, while glycerol-induced suppression on AOX expression was at least partially removed in the triple mutant. However, glucose-induced suppression on AOX expression was still tight even at low glucose concentration of 1%.

It seemed that glucose repression was more strict than glycerol repression on AOX expression in *P. pastoris*. As for glucose repression, it has been widely reported and is mediated by several transcriptional repressors including Mig1, Mig2, and Nrg1^30^,^32^. They usually functioned by regulating glucose-repressed genes involved in metabolism of other carbon sources, gluconeogenesis, Krebs cycle and in respiration, *etc*^30^,^32^. For instance, Mig1 may bind to promoters of several glucose-repressed genes (*GAL1, SUC2, MAL*) and recruit a co-repressor, the Ssn6-Tup1 complex to repress their transcription^30^,^32^. Nrg1 may also bind to promoters of several glucose-repressed genes (*SUC2, HXT2, PCK1*)^30^,^32^. Therefore, they probably bind to promoters of genes involved in methanol utilization in *P. pastoris*.

However, few reports were on glycerol repression mediated by Mig1, Mig2, and Nrg1. In our previous work, we have identified a transcription activator Mit1 of P_*AOX1*_, which was proved to be closely related with different repression effects on AOX expression by glycerol in *P. pastoris* and *H. polymorpha*^20^. Mit1 has a Zn(II)2Cys6-type DNA-binding domain similar to *H. polymorpha* Mpp1, but it shows low identity to Mpp1 and other proteins with undetermined function. Interestingly, expressing Mpp1 in Δ*mit1* cells showed remarkable AOX expression in the presence of glycerol in *P. pastoris*. Thus we analyzed the functions of different domains in Mit1 and verified that the structural dissimilarity between Mit1 and Mpp1 contributed to the differential repression of *AOX1* promoter and *MOX* promoter in the presence of glycerol, respectively, in *P. pastoris* and *H. polymorpha*. As complementing Mpp1 in Δ*mit1* cells derepressed AOX expression in glycerol in *P. pastoris*, Mig1, Mig2 and Nrg1 should not function for various glycerol repressions in different strains. Besides, as Mig1, Mig2 or Nrg1 functioned widely in catabolite repression, they might regulate Mit1 expression by binding to promoter of *MIT1* or interact with Mit1 protein, which further functioned for glycerol repression. They may also bind to promoters of other genes involved in methanol utilization. For catabolite repression, the glucose repression is typically and widely existed in various organisms, thus Mig1, Mig2 and Nrg1 may regulate genes involved in methanol utilization more finely and strictly when cells grown in glucose than in glycerol. However, it still needs substantial works to clarify the different repression mechanisms mediated by glucose and glycerol.

### Overexpression of transcription activators in repressors-deficient strain highly derepressed *AOX1* promoter in Glycerol

**Figure 2:**
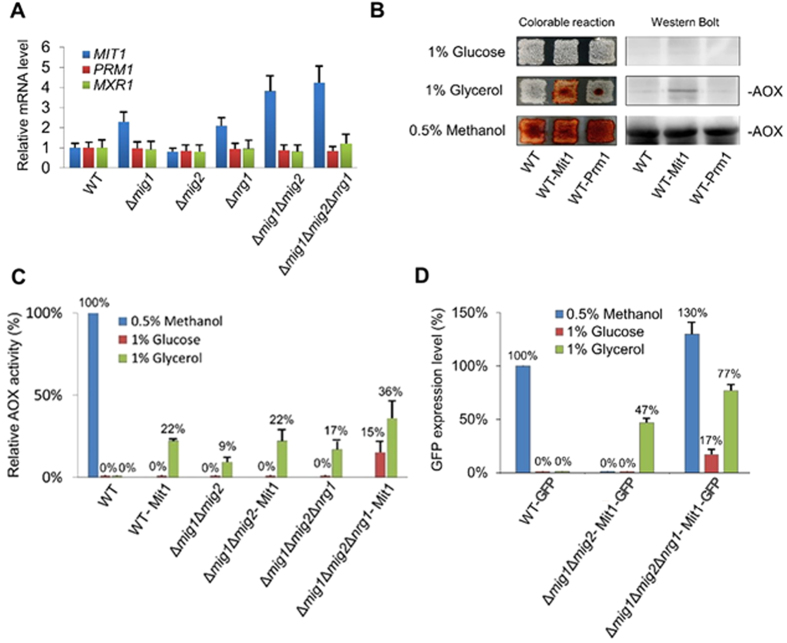
Overexpression of transcription activator Mit in transcription repressors-deficiency strains highly derepressed AOX and GFP expression by P_*AOX1*_. (**A**) Studies on the mRNA expression levels of *MIT1, PRM1* and *MXR1* in WT, Δ*mig1*, Δ*mig2*, Δ*nrg1*, Δ*mig1*Δ*mig2* and Δ*mig1*Δ*mig2*Δ*nrg1* strains when grown in 1% glycerol. Quantitative RT-PCR (qRT-PCR) was performed to determine the levels of mRNA. The mRNA levels were normalized to the levels of *ACT1* mRNA in each sample, and relative to the levels of each gene mRNA in WT. The error bars represent the mean of three biological replicates assayed in duplicate. (**B**) Colorimetric reaction of AOX enzyme assay of WT, WT-Mit1 and WT-Prm1 strains on different carbon sources. Cells were grown for 2 days on YND agar medium and then replica plated onto medium containing different carbon sources. Twelve hours later, AOX activity was visualized by overlaying the AOX activity reaction mixture along with a permeabilizing agent. Western blot detection of AOX protein in WT, WT-Mit1 and WT-Prm1 strains induced on different carbon sources. Original gels/blots are provided in Supplementary Fig. S2. (**C**) Relative AOX activity levels of WT, WT-Mit1, Δ*mig1*Δ*mig2*, Δ*mig1*Δ*mig2*-Mit1, Δ*mig1*Δ*mig2*Δ*nrg1* and Δ*mig1*Δ*mig2*Δ*nrg1*-Mit1 strains cultured in different carbon sources. Cells of each strain were grown for 24 hours in YND medium and transferred into fresh YNB medium supplemented with different carbon sources (0.5% methanol, 1% glucose and 1% glycerol) at an initial OD_600_ of 1.0. Cells were collected every 3 hours and AOX specific activity was measured in cell extracts prepared as described in materials and methods. The highest AOX specific activity of each strain in different carbon sources was presented. (**D**) Relative GFP expression levels of WT-GFP, Δ*mig1*Δ*mig2*-Mit1-GFP, Δ*mig1*Δ*mig2*Δ*nrg1*-Mit1-GFP strains during growth on different carbon sources. Cells of each strain were grown for 24 hours in YND medium and transferred into fresh YNB medium supplemented with different carbon sources (0.5% methanol, 1% glucose and 1% glycerol) at an initial OD_600_ of 1.0. Cells were collected every 3 hours and GFP expression was monitored. The highest GFP expression of each strain in different carbon sources was presented.

As previously reported, transcription activators Mxr1^21^,^22^,^33^,^34^ and Prm1^35^,^36^ played important roles in activating P_*AOX1*_ in response to methanol. We also identified an activator Mit1 participating in P_*AOX1*_ regulation^20^. The three activators were bound to P_*AOX1*_ at different sites and did not interact with each other. However, these factors cooperatively activated P_*AOX1*_ through a cascade^20^. To determine how these three transcriptional activators respond to the loss of the above three transcriptional repressors, we measured mRNA expression levels by qRT-PCR in both wild-type cells and the deletion mutants. When wild-type cells and all the mutants were cultured in glycerol, mRNA expression levels of *MXR1* and *PRM1* remained similar in all the cells (Fig. 2A). The expression level of *MIT1* also remained the same in Δ*mig2* but increased dramatically when either *MIG1* or *NRG1* was deleted, either individually or in the double and triple knockout mutants (Fig. 2A). These results indicated that when cells were grown in glycerol, *MIG1* and *NRG1* repress the expression of *MIT1*.

Then Mit1 was overexpressed under constitutive promoter P_*GAP*_ in wild-type cells and changes in AOX expression were determined when the cells were cultured in different carbon sources. Meanwhile, Prm1 was overexpressed as a control. Mxr1 was not included because its overexpression is toxic^21^. When cells were cultured in glucose, overexpression of Mit1 or Prm1 did not induce AOX expression (Fig. 2B). When cultured in methanol, both overexpression strains and wild-type expressed AOX, as indicated by the colorimetric reaction on the left and the Western blot on the right. Overexpression of Mit1 but not Prm1 induced AOX expression in glycerol, suggesting that a certain amount of the transcriptional activator activity of Mit1 on *AOX1* promoter can be derepressed by glycerol but not by glucose.

Based on these observations, we overexpressed Mit1 under the *GAP* promoter in the double and triple mutants, and measured AOX activity in the medium containing various carbon sources. When grown in 1% glycerol, AOX activity was detected in both the double and triple deletion mutants (Fig. 2C), in accordance with the AOX expression in Fig. 1A and B. When P_*GAP*_-Mit1 was introduced into the double and the treble deletion mutant (Δ*mig1*Δ*mig2*Δ*nrg1*-Mit1, MF1), cells cultured in glycerol achieved 22% and 36% of AOX activity compared to wild-type cells cultured in methanol. As the AOX activity in Δ*mig1*Δ*mig2*Δ*nrg1* cells cultured in glycerol only reached 9% and 17% of wild-type cells in methanol, overexpression of Mit1 had a clear impact on the expression of AOX. To evaluate the potential utility of expressing recombinant proteins in MF1, we measured GFP expression controlled by the *AOX1* promoter. As shown in Fig. 2D, the expression level of GFP in MF1 in glycerol reached 77% of that in wild-type cells induced by methanol. This suggested that the mutant cells induced by glycerol could produce recombinant proteins nearly as efficiently as wild type cells induced by methanol. Moreover, as shown in Fig. 2C and D, repression of glucose on P_*AOX1*_ was more strict than that of glycerol. It seemed that the relative expression level of GFP was much higher than that of AOX in the recombinant strains when grown on glycerol. As is known, methanol usually acts as a strong peroxisome proliferator for *Pichia pastoris*^37^. The synthesized AOX usually localized in peroxisomes to catalyze methanol to formaldehyde, which will be further catabolized to non-toxic substrates via several steps^28^. Therefore, the formation of peroxisomes in *P. pastoris* cells in glycerol may be weaker than that in methanol, which further leads to lower level of AOX content. Differently, the expressed GFP distributed freely in cytoplasm regardless of peroxisome proliferation in various carbon sources.

### Designing novel glucose-glycerol-shift induction strategy for protein production in *P. pastoris* MF1

**Figure 3:**
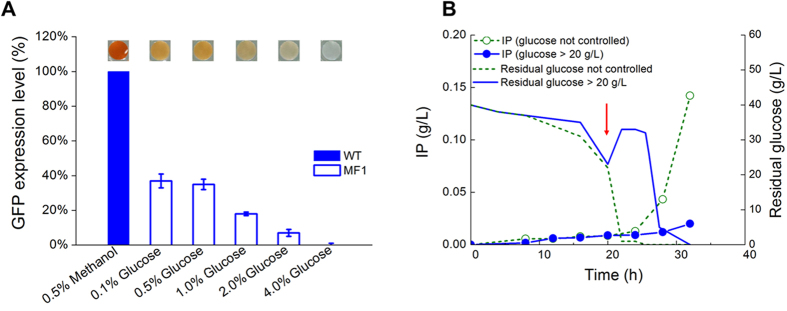
Glucose control for repression and derepression of recombinant protein expression by P_*AOX1*_ in the MF1 and MF1-IP strains. (**A**) Relative AOX activity (colorimetrical assay) and GFP expression levels driven by P_*AOX1*_ in the wild type and MF1 strains grown on different concentration of glucoses (0.1%, 0.5%, 1%, 2%, 4%) and of wild type cells cultured with 0.5% methanol. Cells were transferred to YNB medium with indicated carbon sources and nutritional supplements to a density at OD_600_ of 1.0. Then after 3 h, relative AOX activity and GFP expression levels were analyzed. The residual glucose levels were detected after 3 h culture, which was 0.007%, 0.41%,0.89%, 1.89%, 3.83% for the original levels of 0.1%, 0.5%, 1%, 2%, 4%, respectively. Deep color indicated high AOX activity. Single copy of GFP was expressed under P_*AOX1*_ for either strain. Detailed culture and analysis methods were described in Materials and methods. (**B**) Residual glucose and insulin precursor (IP) expression of the MF1-IP strain cultured in a 5-L bioreactor with different glucose control strategies. The arrow indicated the starting point (21 h) of glucose fed-batch phase. Residual glucose controlled at not lower than 20 g/L effectively inhibited IP expression driven by P_*AOX1*_.

One major advantage of P_*AOX1*_ is the tight response of methanol^3^, and this makes P_*AOX1*_ controllable to express heterologous protein toxic to host^5^. Also, induced protein expression for the high cell density fermentation is usually favored. Thus, for the methanol-independent strain MF1, it is worth to developing an induction manner for heterologous protein expression. In order to find out the way making P_*AOX1*_ controllable, the intensity of P_*AOX1*_ in different concentrations of glucose was determined by AOX activity assay reflected by colorimetrical assay^23^. As shown in Fig. 3A, AOX activity decreased with the increase of glucose concentration. Extremely low level of AOX activity was detected in the MF1 in the presence of 2% glucose, and almost no AOX activity was detected when the strain cultured in 4% glucose. The GFP expression results accorded with those of AOX activity. Moreover, residual glucose concentrations in cultures with initial glucose levels of 0.5%, 1.0%, 2.0% and 4.0% were still high enough for glucose repression at the time point for AOX and GFP detections. These results proved that P_*AOX1*_ in the MF1 could be highly suppressed at glucose concentration over 2%.

For protein expression in the wild-type, the typical fermentation process usually follows a three-stage strategy^11^: (1) a batch phase for biomass growth with glycerol; (2) a fed-batch phase for further biomass enhancement with glycerol; and (3) an optional methanol-induced adaptation (transition) phase, which is followed by a production phase in fed-batch mode. This strategy makes *P. pastoris* grow in a fed-batch manner to a high cell density in order to obtain a high productivity^38^ and efficiently express heterologous protein toxic to host^5^.

According to the results of Figs 2D and 3A, P_*AOX1*_ in the MF1 cells could be suppressed by high concentration of glucose, derepressed by glycerol or low concentration of glucose. Thus, a novel methanol-independent fermentation process was then designed by a three-stage strategy: (1) a batch phase for biomass growth with initial glucose of 40 g/L, suppressing P_*AOX1*_; (2) a fed-batch phase for further biomass enhancement with glucose feeding to maintain residual glucose not lower than 20 g/L, suppressing P_*AOX1*_; and (3) glucose used up and glycerol feeding induced the expression of heterologous protein driven by P_*AOX1*_ as the carbon source without methanol in a fed-batch mode.

In order to determine the fermentation process of glucose growth phase, the MF1 producing a secreted insulin precursor (IP) was constructed and cultured in a 5-L bioreactor by different glucose feeding strategies. As shown in Fig. 3B, the expression of IP was inhibited in glucose fed-batch phase when residual glucose was controlled above 20 g/L. Oppositely, the expression of IP increased dramatically after glucose in bioreactor exhausted. These results suggested that expression of heterologous proteins in the MF1 could be controlled by altering glucose concentrations. Therefore, the P_*AOX1*_ promoter could be turned off/on by glucose-glycerol-shift in our new methanol-independent *Pichia* system.

### Comparisons of the novel engineered methanol-free system with the wild type methanol-induced system of *P. pastoris* during fermentation

Although the novel induction strategy of glucose-glycerol-shift was proved to be feasible, the cell growth and metabolism in MF1 must be different from the WT because genes were reformed and carbon sources during different phases were altered. Thus we optimized the fermentation bioprocess (DO level of 30%~50%, pH 3.5, induction biomass of 200 g/L, glycerol feeding rate of 15.5 mL/L/h) for this new system (Supplementary Fig. S3).

**Figure 4:**
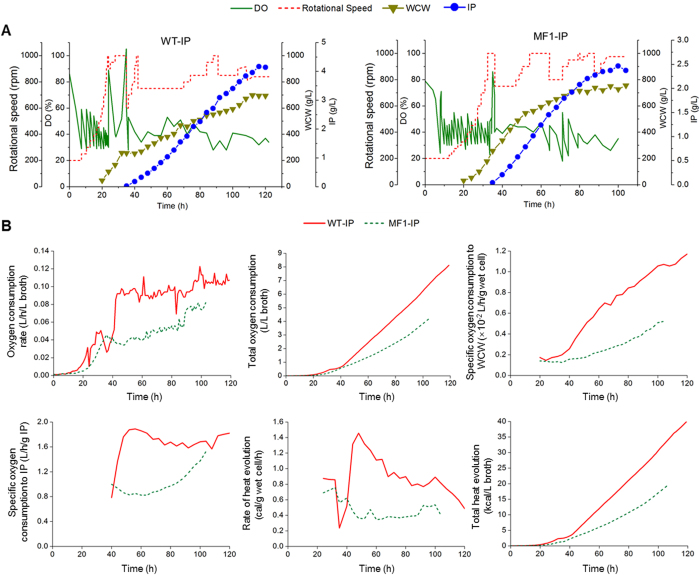
Comparsion of fermentation process for recombinant protein expression in the wild type and the MF1-IP strains. (**A**) Time profiles of dissolved oxygen (DO), rotation speed, wet cell weight (WCW), insulin precursor (IP) in a 5-L bioreactor fermentation; (**B**) Time profiles of oxygen consumption rate, total oxygen consumption, specific oxygen consumption to biomass, specific oxygen consumption to IP production, rate of heat evolution, total heat evolution in a 5-L bioreactor fermentation. The details of measurement and calculation methods were described in the supplementary file.

Then, the whole fermentation processes were evaluated between the engineered methanol-free system and the wild type methanol-induced system under their specific optimal culture conditions. As shown in Fig. 4A, Cell growth of both strains were normal. The expression level of IP reached 2.46 g/L in the MF1-IP, which was 58.6% of the WT-IP (4.20 g/L). Transition from glucose to glycerol in the MF1-IP was much easier than that from glycerol to methanol in the WT-IP, which ascribed to that, excess methanol was usually toxic to cells and easily caused irreversible metabolic adaptation. The oxygen consumption rate of the WT-IP was two times higher than that of the MF1-IP during the induction phase (Fig. 4B). The mixture of pure oxygen and air was used as in-gas at 42 h in the WT-IP, when the WCW reached 250 g/L. However, the mixture of pure oxygen and air was only used at 65 h in the MF1-IP, when the WCW was about 650 g/L. The total oxygen consumption of the MF1-IP reached 3.94 L/L fermentation broth, 51.5% lower than that of the WT-IP during the whole fermentation process. The specific oxygen consumption to WCW of the MF1-IP reached 0.0052 L/h/g wet cell, 55.5% lower than that of the WT-IP. The specific oxygen consumption to IP of the MF1-IP reached 1.52 L/h/g IP, 16.3% lower than that of the WT-IP. The total heat evolution of the MF1-IP reached 19.33 kcal/L fermentation broth, 51.5% lower than that of the WT-IP. The highest rate of heat evolution in induction phase of the MF1-IP reached 0.51 cal/g wet cell/h, 65.7% lower than that of the WT-IP. Thus, it will save a lot of cooling costs especially for the industrial use.

**Figure 5:**
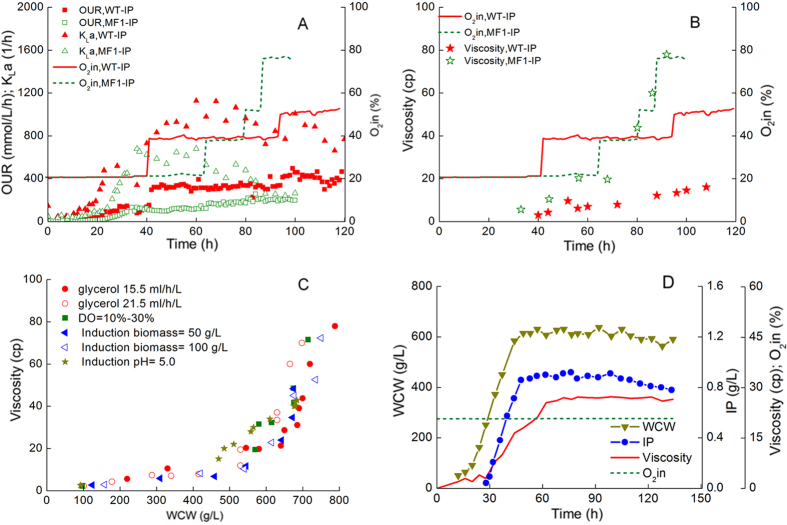
Relationship clarification of oxygen consumption, broth viscosity and biomass accumulation during fermentation of the MF1-IP strains. *OUR* (oxygen uptake rate), *k*_*L*_*a* (mass-transfer coefficient) *vs*. oxygen volume content of the in-gas (**A**), viscosity *vs*. oxygen volume content of the in-gas (**B**), relationship between wet cell weight (WCW) and viscosity (**C**) of the MF1-IP and WT-IP strains in fed-batch fermentation. (**D**) chemostat culture of the MF1-IP strain with a dilution rate of D = 0.02 1/h. Culture and analysis methods were described in Materials and methods.

It is curious that the MF1-IP also needed pure oxygen inlet after 65 h, despite the oxygen consumption of it was only half of that of the WT-IP. To clarify this, *OUR, k*_*L*_*a* and viscosity were analyzed. According to Fig. 5A, in induction phase, the *OUR* of the WT-IP reached 464.7 mmol/Lbroth/h, which reached 211% of that of the MF1-IP. This result accorded with oxygen consumption, mainly ascribed to huge oxygen requirement and heat release of methanol oxidation. Moreover, the *k*_*L*_*a* of the WT-IP increased faster than that of the MF1-IP during the growth phase because the rotation speed of the WT-IP increased faster. The *k*_*L*_*a* of the WT-IP was much higher than that of the MF1-IP in induction phase, which was kept between 729.9 1/h and 1128.1 1/h. Interestingly, the *k*_*L*_*a* of the MF1-IP kept declining rapidly during the induction phase, which decreased from 678.2 1/h to 242.5 1/h despite that the *OUR* increased. Accordingly, the oxygen concentration in the gas inlet kept increasing to 77% in the late phase of fermentation. Therefore, the oxygen transfer in bioreactor became worse and worse with MF1-IP culture going on. As shown in Fig. 5B, the viscosity of fermentation broth of the MF1-IP increased from 19.7 cp to 80 cp, going with the increase of the oxygen concentration in the gas inlet. However, the viscosity of the WT-IP remained below 20 cp. The results indicated that poor oxygen transfer in the MF1-IP fermentation was mainly due to the high broth viscosity.

Then we need to find out the reason for the sharp increase of viscosity during the late phase. Thus the relationship between broth viscosity and WCW of different batches of the MF1-IP was analyzed. As shown in Fig. 5C, the viscosity went up with the increase of WCW consistently. It was worth noticing that the viscosity increased rapidly after WCW was over 650 g/L. Therefore, WCW of the whole cultivation should be controlled below 650 g/L to avoid excessive viscosity. To verify the effect of WCW on viscosity, chemostat cultivation was carried out. The WCW was maintained at about 600 g/L, and the dilution rate was controlled at 0.02 1/h. The viscosity was maintained at about 25 cp during the whole culture process (Fig. 5D) and pure oxygen was no longer needed. These results indicated that high viscosity could be avoided when WCW was controlled at 600 g/L.

As for the WT-IP, its fermentation process has been optimized for many years in our group and is the highest IP production reported so far, which has been applied in a biopharmaceutical company. Although the IP production by MF1-IP was lower than by WT-IP here, it is still a high titer and is higher than other reported results even from the wild-type methanol strategies (Table 1). To further improve the insulin production, two aspects could be considered. On one hand, expression strains owning higher gene dosage can be constructed and screened^39^; and correspondingly, overexpression of unfolded protein response activators, protein foldases or chaperons may further facilitate protein secretion and increase the production^40^. On the other hand, bioprocess control should be further optimized. An alternative feeding strategy with mixed glycerol and other fermentative carbon sources may be investigated to regulate cell growth and reduce the viscosity during late culture phase. Moreover, for the potential industrial use, semi-continuous culture may be involved^41^ so that broth viscosity will be reduced and the final product will be accumulated intermittently. Overall, the safe substrates and process will promote it for potential industrial use especially in food and drug area. This mutant and bioprocess represent a safe and efficient alternative to the traditional methanol-based expression system.

## Materials and Methods

### Plasmids and strains

Standard procedures for the manipulation of plasmid DNA were described before^23^. Mig1 and Mig2 subcellular localization strains were firstly constructed by expressing a *GFP-MIG1* fusion gene under a constitutive *GAP* promoter in the wild type strain *P. pastoris* GS115. Three strains of Δ*mig1*, Δ*mig2* and Δ*nrg1* were constructed by double crossover homologous recombination to evaluate the repression roles of the transcription factors Mig1, Mig2 and Nrg1 in regulating P_*AOX1*_. Then the Δ*mig1* and Δ*mig2* strains were crossed and diploid hybrids^34^, which resulted in a Δ*mig1*Δ*mig2* double-deletion mutant. Furthermore, a Δ*mig1*Δ*mig2*Δ*nrg1* treble-deletion strain was constructed by the deletion of the *NRG1* gene in the Δ*mig1*Δ*mig2* double-deletion strain. Two strains overexpressing transcription activator genes *MIT1* and *PRM1* were constructed to test their promotion effects on P_*AOX1*_ and designated as WT-Mit1 and WT-Prm1, respectively. The Mit1 was then overexpressed in the strains of Δ*mig1*Δ*mig2 and* Δ*mig1*Δ*mig2*Δ*nrg1*, respectively, and generated Δ*mig1*Δ*mig2*-Mit1 and Δ*mig1*Δ*mig2*Δ*nrg1*-Mit1 (MF1). The green fluorescent protein was overexpressed in the wild-type, Δ*mig1*Δ*mig2-*Mit1 and MF1, which resulted in WT-GFP, Δ*mig1*Δ*mig2-*Mit1-GFP and MF1-GFP, respectively. A gene encoded for insulin precursor (IP)^42^ was overexpressed in the MF1 strain. Strains with multiple gene copies were screen and the strain produced the highest IP level was selected as the final IP producing strain MF1-IP, which was used for comparsion with the IP production ability in the wild-type (WT-IP)^42^. The strains with genotype information used for this study are listed in Table 2. The primers and plasmids used for this study are listed in Supplementary Tables S1 and S2, respectively. Detailed information on construction of strains are provided in the supplementary file.

### Miscellaneous methods

Enzyme assays of alcohol oxidase and fluorescence measurement were adapted from previous studies^23^,^25^. The alcohol oxidase (AOX) activity was assayed spectrophotometrically with peroxidase and 2, 2’-azino-di-(3-ethylbenzthiazoline sulfonate) (ABTS) as described previously^43^. A unit of AOX represents 1 μmol of product/min/mg of protein at 30 °C. Samples with methanol omitted was run as blanks. The AOX colony assay was performed as previously described^44^. The quantification of GFP expression was monitored by a multi-mode microplate reader (Biotek). The excitation wavelength was 485 nm when the emission wavelength was 515 nm. The quantification of GFP expression levels driven by P_*AOX1*_ in WT and *P. pastoris* MF1 strains was monitored. Cells were cultured in yeast nitrogen base medium (0.67% YNB) containing 1% glucose (YND), 1% glycerol (YNG) or 0.5% methanol (YNM), respectively. Cells of each strain were pre-grown overnight in YND medium and transferred into the YND, YNG and YNM media at an initial OD_600_ of 1.0. The quantification levels of GFP expression of each strain were the highest during the growth process respectively. For western blot analysis, yeast cells were first grown overnight in YPD medium to stationary phase. Aliquots of the stationary culture were used to inoculate an appropriate volume of YND medium containing any necessary supplements. Cells were grown to an optical density at 600 nm (OD_600_) of approximately 2~6 and then collected, washed three times by sterile water. For induction purpose, cells were transferred to YNB media with indicated carbon sources and nutritional supplements to a final optical density at OD_600_ of 1.0. The cells were grown with vigorous shaking at 30 °C for 10 h, cell pellets were harvested and then processed for cell extract preparation and western blot analysis as described previously^23^. The differential expression of three transcriptional activator genes *MIT1, MXR1* and *PRM1* in WT, Δ*mig1*, Δ*mig2*, Δnrg1, Δ*mig1*Δ*mig2* and Δ*mig1*Δ*mig2*Δnrg1 were verified by RT-PCR. Cells were prepared as described in western blot analysis except that cells were induced in glycerol for 6 hours. RT-PCR was carried out as described previously^19^, and the primers was designed by Primer Express software (Applied Biosystems, Foster City, CA) and listed in Supplementary Table S3. The cycle threshold values (CT) were determined and the relative fold differences were calculated by the 2^−ΔΔCT^ method^45^, using actin 1 (*ACT1*) as the endogenous reference genes^46^. The online pH and dissolved oxygen (DO) in bioreactor was measured by an online pH and dissolved oxygen sensor (Mettler Toledo). Offline pH was detected by a pH detector (Mettler Toledo). The measurement of wet cell weight (WCW) followed: 1 mL fermentation broth was transferred into an 1.5 mL Eppendoff tube; the tube with fermentation broth was centrifuge for 3 min at 8000 *g*, and supernatant was discarded; weight the total weight of the tube and precipitation; the weight of the tube was subtracted from the weight of the tube and precipitation; the weight left was the WCW, g/L. The IP titer was determined by HPLC^42^. The measurement of viscosity was monitored by a digital viscometer (DV-2+ Pro, Shanghai Nirun Intelligent Technology Co., Ltd.), the temperature control and rotor speed for measurement was 10 °C and 200 rpm, and the sample volume was 15 mL.

### Bioreactor fed-batch and chemostat fermentation

For fed-batch culture, two milliliters of *P. pastoris* MF1-IP cells (OD_600_ = 7.0–8.0) stored in −80 °C were inoculated directly into 500-mL shake flasks containing 100 mL YND medium as the first seed culture. After cultivated at 30 °C and 200 rpm for 36 h, 37.5 mL first seed culture was inoculated into a 1 L shake flask containing 300 mL YND to obtain the second seed culture. In 5 L bioreactor, culture was started with a glucose growth phase with 3.5 L basal salt medium, which contains (per liter): 13 mL H_3_PO_4_ (85%), 0.82 g CaSO_4_, 18.2 g K_2_SO_4_, 14.9 g MgSO_4_·7H_2_O, 10.6 g KOH, 13.2 g (NH_4_)_2_SO_4_, 40.0 g glucose, 0.33 mL antifoam, and 4.5 mL PTM1 trace salts. The PTM1 trace salts stock solution contains (per L): 6.0 g CuSO_4_·5H_2_O, 0.08 g NaI, 3.0 g MnSO_4_·H_2_O, 0.2 g Na_2_MoO_4_·2H_2_O, 0.02 g H_3_BO_3_, 0.5 g CoCl_2_, 20.0 g ZnCl_2_, 65.0 g FeSO_4_·7H_2_O, 0.2 g biotin, and 5.0 mL of H_2_SO_4_. During glucose growth phase, 50% (w/v) glucose containing 12 mL PTM1 per liter was fed with a feeding rate of 50 mL/h. After wet cell weight (WCW) reaching about 200 g/L and glucose was almost exhausted, glucose growth phase was finished and then switched to glycerol fed-batch phase. Feeding medium was 50% (w/v) glycerol containing 12 mL PTM1 per liter. During the fermentation process, the temperature was maintained at 30 °C. The pH was kept at 5.0 by adding 25% ammonia solution, and the dissolved oxygen (DO) was kept above 30%. The WT-IP strain was cultured as a control following the previously described method^42^, for which, cells grew in glycerol to WCW of about 200 g/L and then switched to methanol feeding after glycerol exhausted. For chemostat culture, second seed of 600 mL was inoculated into 3 L medium in a 5 L stirred tank bioreactor. Continuous cultures were maintained at a working volume of 3 L at 30 °C with a minimum of 30% DO. Batch cultivations were performed in the basal salt medium. The pH was controlled at 5.0 in glucose phase and 3.5 in glycerol phase with 25% ammonium hydroxide. After approximately 24 h, the batch finished and the chemostat culture started with a dilution rate of D = 0.02 1/h. The chemostat medium contains (per liter): 13 mL H_3_PO_4_ (85%), 0.82 g CaSO_4_, 18.2 g K_2_SO_4_, 14.9 g MgSO_4_·7H_2_O, 10.6 g KOH, 13.2 g (NH_4_)_2_SO_4,_ 300 g glycerol, 0.33 mL antifoam, and 4.5 mL trace salt stock solution. The continuous culture was performed for at least five resident times to reach steady-state conditions. The working volume was kept constant by balancing medium feeding and broth removing.

### Oxygen consumption, heat evolution, *OUR* and *k*_*L*_ *a*

The CO_2_ and O_2_ concentration of in-gas and off-gas were determined by exhaust gas analyzer (FerMac 368, Electrolab), while the mass flow of in-gas and off-gas was monitored by a mass air flow meter.

The oxygen consumption rate was calculated by the equation below:


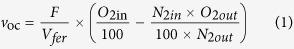


where ν_oc_ is oxygen consumption rate, L/h/L fermentation broth; *F* is volume flow under standard state (temperature is 0 °C, pressure is 1 atm) of the gas, L/h; *O*_*2in*_, *O*_*2out*_ is volume content of the in-gas and off-gas, %, respectively; *V*_fer_ is volume of the fermentation broth, L; *N*_2in_, *N*_2out_ is the nitrogen concentration in the gas inlet and gas outlet, respectively.

The specific oxygen consumption to WCW and the specific oxygen consumption to IP were calculated by the following equation:


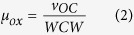



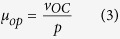


where *μ*_*ox*_ is specific oxygen consumption to *WCW*, L/h/g wet cell; *WCW* is wet cell weight, g/L; *μ*_*op*_ is specific oxygen consumption to IP, L/h/g IP; *p* is concentration of IP in supernatant, g/L fermentation broth;

The total heat released was calculated by the following equation^47^:


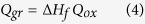


where *Q*_*gr*_ is total heat released, kcal/L fermentation broth; ∆*H*_f_ = 0.11 kcal/mmol O_2_ is a proportionality constant; *Q*_*ox*_ is total oxygen consumption (mmol/L fermentation broth).

The rate of the heat evolution was calculated by the following equation^48^:


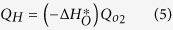



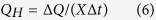


where *Q*_H_ is specific rate of heat evolution, cal/g wet cell/h; −∆*H*_O_^*^ = 106 cal/mmol O_2_ is heat of combustion per available electron equivalent; 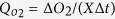 is specific rate of respiration, mmol O_2_/g wet cell/h.

*OTR* were assumed to equal *OUR* and were calculated as follows:


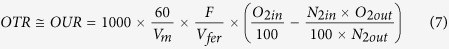


where *OTR* is the oxygen transfer rate, mmol/L/h; *OUR* is the oxygen uptake rate, mmol/L/h; *V*_*m*_ is the molar volume of gas, 22.4 L/mol; *F* is volume flow under standard state (temperature is 0 °C, pressure is 1 atm) of the gas, L/h; *V*_*fer*_ is the volume of fermentation broth; *O*_*2in*_, *O*_*2out*_ is the oxygen concentration in the gas inlet and gas outlet, %, respectively; *N*_2in_, *N*_2out_ is the nitrogen concentration in the gas inlet and gas outlet, respectively.

*K*_*L*_*a* was determined by the equation below:


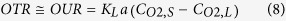


where *C*_*O2*_,_*S*_ is the oxygen concentration at saturation in the liquid phase, mmol/L; *C*_*O2*_,_*L*_ is the oxygen concentration in the liquid phase, mmol/L; *K*_*L*_*a* is the mass-transfer coefficient, 1/h.

Oxygen concentration at saturation under fermentation conditions varies with the fermenter overhead pressure and oxygen concentration in gas, and it can be calculated using Henry’s law^49^:


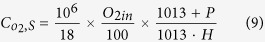


where *P* is the fermenter overhead pressure, mbarg; *H* is Henry’s law proportionality constant, 4.75 × 10^4^ atm/mol/mol (water at 30 °C).

## Additional Information

**How to cite this article:** Wang, J. *et al*. Methanol-Independent Protein Expression by *AOX1* Promoter with *trans*-Acting Elements Engineering and Glucose-Glycerol-Shift Induction in *Pichia pastoris. Sci. Rep.* **7**, 41850; doi: 10.1038/srep41850 (2017).

**Publisher's note:** Springer Nature remains neutral with regard to jurisdictional claims in published maps and institutional affiliations.

## Supplementary Material

Supplementary Information

## Figures and Tables

**Table 1:** Insulin precursor productions by recombinant *P. pastoris*.

Strain type (copy)	Induction carbon source	Product in culture supernatant^a^ (g/L)	Reference
Mut^+/s^ (6–8)	Methanol	1.50	50
Mut^s^ (nd)^b^	Methanol	0.22	51
Mut^s^ (11)	Methanol	0.30	52
Mut^s^ (11)	Methanol	0.25	53
Mut^+^ (nd)^c^	Methanol	3.60	42
Mut^+^ (12)	Methanol	0.18	54
Mut^+^ (~2)^d^	Methanol	3.84	55
Mut^+^ (7.2)	Methanol	0.38	56
Mut^+^ (nd)	Methanol	0.062	57
WT-IP (Mut^+^ (nd)^e^)	Methanol	4.20	This study
MF1-IP (Mut^+^ (nd))	Glycerol	2.46	This study

Mut^+^, methanol utilization plus phenotype, which owns intact *AOX1* and can grow normally in methanol; Mut^s^, methanol utilization slow phenotype, which owns deficient *AOX1* but intact *AOX2* and grow slowly in methanol. ^a^Concentrations were determined by reverse phase HPLC from culture supernatant; ^b^nd; not determined; ^c,e^Copy number not experimentally determined; ^d^Copy number not experimentally determined. Survival at zeocin concentrations up to 0.5 mg/mL indicated a copy number of 2. WT-IP, the strain for insulin precursor expression constructed based on the wild type; MF1-IP, the strain for insulin precursor expression constructed based on the recombinant Δ*mig1*Δ*mig2*Δ*nrg1-*Mit1-IP (MF1). Strain constructions were shown in Materials and methods.

**Table 2:** Strains used in this study.

Strain	Genotype	Source
***E**. **coli***		
Top 10	F’[*lacI*^q^ Tn*10* (Tet^r^)] *mcrA* Φ80*lacZ* ΔM15 Δ*lac X74 deoR recA1*	Invitrogen
***P**. **pastoris***		
GS115 (Wild-type)	*his4*	Invitrogen
GS115/pGLM1	GS115 P_*GAP*_:: pGLM1 (P_*GAP*_-GFP-Mig1 *HIS4, Sh ble*)	This study
GS115/pGLM2	GS115 P_*GAP*_:: pGLM2 (P_*GAP*_-GFP-Mig2 *HIS4, Sh ble*)	This study
Δ*mig1*	GS115 *mig1*Δ*::Sh ble his4*	This study
Δ*mig2*	GS115 *mig2*Δ*::KAN his4*	This study
Δ*nrg1*	GS115 *nrg1*Δ*::hph his4*	This study
Δ*mig1*Δ*mig2*	GS115 *mig1*Δ*::Sh ble mig2*Δ*::KAN his4*	This study
Δ*mig1*Δ*mig2*Δ*nrg1*	GS115 *mig1*Δ*::Sh ble mig2*Δ*::KAN nrg1*Δ*::hph his4*	This study
WT-Mit1	GS115 P_*GAP*_::pP6GM1 (P_*GAP*_*-*Mit1 *HIS4, Bla*)	This study
WT-Prm1	GS115 P_*GAP*_::pPGP1 (P_*GAP*_-Prm1 *HIS4, KAN*)	This study
Δ*mig1*Δ*mig2-*Mit1	GS115 *mig1*Δ*::Sh ble mig2*Δ*::KAN P*_*GAP*_::pP6GM1 (*P*_*GAP*_*-*Mit1 *HIS4, Bla*)	This study
Δ*mig1*Δ*mig2*Δ*nrg1-*Mit1 (MF1)	GS115 *mig1*Δ*::Sh ble mig2*Δ*::KAN nrg1*Δ*::hph P*_*GAP*_::pP6GM1 (*P*_*GAP*_*-*Mit1 *HIS4, Bla*)	This study
WT-GFP	GS115 *his4*::pP-GFP (P_*AOX1*_*-*GFP *HIS4*)	This study
Δ*mig1*Δ*mig2-*Mit1*-*GFP	GS115 *mig1*Δ*::Sh ble mig2*Δ*::KAN P*_*GAP*_::pP6GM1 (*P*_*GAP*_*-*Mit1 *Bla*) *his4::*pP-GFP (*P*_*AOX1*_*-*GFP *HIS4*)	This study
Δ*mig1*Δ*mig2*Δ*nrg1-*Mit1-GFP (MF1-GFP)	GS115 *mig1*Δ*::Sh ble mig2*Δ*::KAN nrg1*Δ*::hph P*_*GAP*_::pP6GM1 (*P*_*GAP*_*-*Mit1 *Bla*) *his4::*pP-GFP (*P*_*AOX1*_*-*GFP *HIS4*)	This study
WT-IP	GS115 *his4::*pPIC9K/IP (*P*_*AOX1*_*-*IP *HIS4*)	This study
Δ*mig1*Δ*mig2*Δ*nrg1-*Mit1-IP (MF1-IP)	GS115 *mig1*Δ*::Sh ble mig2*Δ*::KAN nrg1*Δ*::hph P*_*GAP*_::pP6GM1 (*P*_*GAP*_*-*Mit1 *Bla*) *his4::*pPIC9K/IP (*P*_*AOX1*_*-*IP *HIS4*)	This study
