# Supplementary material for: Methanol-Independent Protein Expression by AOX1 Promoter with trans-Acting Elements Engineering and Glucose-Glycerol-Shift Induction in Pichia pastoris
Source: Sci Rep. 2017 Feb 2;7:41850. doi: 10.1038/srep41850 (PMC5288789; doi:10.1038/srep41850)
Supplement: Supplementary Information [file srep41850-s1.pdf]

Supplementary Information

Methanol-Independent Protein Expression by *AOX1* Promoter with *trans*-Acting Elements Engineering and Glucose-Glycerol-Shift Induction in *Pichia pastoris*

Jinjia Wang[1], Xiaolong Wang[1], Lei Shi[1], Fei Qi[1], Ping Zhang[1], Yuanxing Zhang[1,2], Xiangshan Zhou[1,*], Zhiwei Song[3,4,*], Menghao Cai[1,*]

[1] State Key Laboratory of Bioreactor Engineering, East China University of Science and Technology, 130 Meilong Road, Shanghai 200237, China

[2] Shanghai Collaborative Innovation Center for Biomanufacturing, 130 Meilong Road, Shanghai 200237, China

[3] Bioprocessing Technology Institute, 20 Biopolis Way, #06-01 Centros, Singapore

[4] Department of Biochemistry, Yong Loo Lin School of Medicine, National University of Singapore, 21 Lower Kent Ridge Road, Singapore

*Corresponding author. Tel./fax: +86-21-64253306.

E-mail address: cmh022199@ecust.edu.cn (M. Cai); xszhou@ecust.edu.cn (X. Zhou); song_zhiwei@bti.a-star.edu.sg (Z. Song)

Construction of strains.

Standard procedures for the manipulation of plasmid DNA were described before[1]. ***(i) Mig1 and Mig2 subcellular localization strains.*** The sequence between the *Asu*II and *Not*I sites of pGAPZα A (Invitrogen) was replaced with a 743 bp *GFP* DNA (*Asu*II-*Not*I) amplified by PCR from pP-GFP (a vector capable of expressing a green fluorescent protein [GFP] under the control of P_{AOX1})[2] using primers GFP1 and GFP2, which included restriction sites for *Asu*II and *Not*I, respectively. Next, the resulting plasmid pGAP-GFP was further modified by replacing the sequence between the *Not*I and *Sal*I sites with a 1,355 bp *MIG1* DNA (*Not*I- *Sal*I) amplified by PCR from the genomic DNA of *P. pastoris*, thus generating a plasmid pGLM1. Finally, the plasmid pGLM1 was transformed by electroporation into wild-type *P. pastoris* strain GS115. Zeocin resistance transformants were isolated on YPD medium supplemented with 0.1 mg/ml zeocin. The method of construction of the GS115/pGLM2 strain was the same as that used for the GS115/pGLM1 strain. Both of the transformants were used to observe the subcellular localization of GFP-Migl and GFP-Mig2 under repressing (glucose or glycerol) or inducing (methanol) conditions, respectively. ***(ii) Δmig1, Δmig2 and Δnrg1 strains.*** To knock out the *MIG1* gene with the zeocin resistance gene *Sh ble* as a marker, we first constructed plasmid pUC18-MIG1-del, a *MIG1* deletion vector. This deletion vector was constructed as follows: a 728 bp fragment containing sequences of the upstream region of *MIG1* was amplified by PCR using genomic DNA of *P. pastoris* as a template with *Pyrobest* DNA polymerase (TaKaRa, Japan) and the primers for this PCR, MI1U1 and MI1U2, carried restriction sites for *Eco*RI and *Bam*HI, respectively. The upstream region fragment was inserted into *Eco*RI/*Bam*HI-digested plasmid pUC18 (Invitrogen), yielding pMIG1Up. Next, a 1011 bp fragment consisting of the downstream region of *MIG1* was amplified by PCR with the primers MI1D1 and MI1D2 carrying restriction sites for *Sal*I and *Sph*I, respectively. The downstream region fragment was inserted into *Sal*I/*Sph*I-digested plasmid pUC18 (Invitrogen), yielding pMIG1Down. Moreover, to obtain the zeocin resistance gene sequence with its own promoter and terminator, a 1,321 bp fragment was amplified by PCR using the plasmid pPICZ A (Invitrogen) as a template with primers Zeo1 and Zeo2, which included restriction sites for *Bam*HI and *Sal*I, respectively. Then the fragment was cloned into *Bam*HI/*Sal*I-digested vector pMIG1Up to create vector pMIG1Up-*ble*. A 2.05-kb DNA fragment by digesting this plasmid with *Eco*RI and *Sal*I was inserted into *Eco*RI-*Sal*I-digested pMIG1Down, thus generating a deletion vector for *MIG1* (pUC18-MIG1-del). The deletion cassette was released from pUC18-MIG1-del as a 3.06-kb *Eco*RI-*Sph*I-digested fragment and transformed by electroporation into WT *P. pastoris* strain GS115. Zeocin resistance transformants were

isolated on YPD (1% yeast extract, 2% peptone and 1% glucose) medium supplemented with 0.1 mg/ml zeocin. The correct integration of the deletion cassette into the genome and replacement of a 930 bp fragment near the upstream region of the full *MIG1* open reading frame (ORF) (1335bp) in the transformants were confirmed by PCR analysis and DNA sequencing. Knockout of the *MIG2* gene was the same as that used for the *MIG1* gene other than using the G418 resistance gene *KAN* as a marker, and the gene sequence with its own promoter and terminator (1,517 bp) was amplified by PCR using the plasmid pPIC3.5K (Invitrogen) as a template with primers Kan1 and Kan2. The full ORF of *MIG2* was totally replaced. Knockout of the *NRG1* gene was the same as that used for the *MIG1* gene other than using the hygromycin resistance gene *hph* as a marker, and the gene sequence with its own promoter and terminator (1,648 bp) was amplified by PCR using the plasmid pRDM054[3] (kindly provided by Prof. Suresh Subramani, University of California, San Diego) as a template with primers Hyg1 and Hyg2. The full ORF of *NRG1* was also totally replaced. ***(iii) Δmig1Δmig2 double-deletion strain.*** The Δ*mig1* and Δ*mig2* strains were crossed and diploid hybrids[4] were selected on YPD medium supplemented with zeocin (0.2 mg/ml) and G418 (1 mg/ml). After sporulation, the Δ*mig1*Δ*mig2* double-deletion mutant was selected among spore progeny on the same medium, and confirmed for the presence of *MIG1* and *MIG2* deletion cassettes as detailed above for single-deletion mutants. Mating and sporulation techniques were performed using established procedures[3]. ***(iv) Δmig1Δmig2Δnrg1 treble-deletion strain.*** We constructed the Δ*mig1*Δ*mig2*Δ*nrg1* strain by the deletion of the *NRG1* gene in the Δ*mig1*Δ*mig2* double-deletion strain. The treble-deletion strain was isolated by the gene replacement method in the same way as that used for the Δ*nrg1* single-deletion strain. ***(v) Strains overexpressing Mit1p and Prm1p.*** Constitutively expression Mit1p in yeast, the *MIT1* gene was cloned by PCR using GS115 genome as template with *Eco*RI-MIT1-5 and MIT1-3-XhoI primers. Then the resulting fragment was digested by *Eco*RI + *Xho*I and subsequently inserted into pGAPZA (Invitrogen) and resulted the plasmid pGGM1. Subsequently the pGAP-MIT1 fragment was realized from pGGM1 by *Bgl*II +*Xho*I, and subsequently inserted into *Bgl*II and *Xho*I of pPIC6A (Invitrogen), resulting plasmid pP6GM1. Then the pP6GM1 was linearized by *Bln*I and then transformed to WT, Δ*mig1*Δ*mig2* and Δ*mig1*Δ*mig2*Δ*nrg1* by electroporation, respectively. The transformants were screened by 3 mg/ml blasticidin, resulting strains designated WT-Mit1, Δ*mig1*Δ*mig2*-Mit1 and Δ*mig1*Δ*mig2*Δ*nrg1*-Mit1. The yeast strain constitutively expressing Prm1p was constructed as follows. The *PRM1* gene was amplified from GS115 genome with *Sfu*I-PRM1-5 + PRM1-3-*Xho*I primers. After digested by *Sfu*I and *Xho*I, the fragment was inserted into *Sfu*I and *Xho*I of

pGAPZA, resulting pGGP1. The pGGP1 was linearized by *Bln*I and then transformed to WT strain by electroporation. The transformants were screened by 0.1 mg/ml zeocin, resulting strains designated WT-Prm1. ***(vi) GFP and insulin precursor expression strains.*** To easily describe the following procedure, we then named the strain of Δ*mig1*Δ*mig2*Δ*nrg1*-Mit1 as MF1, based on which, the following strains were constructed. Firstly, pP-GFP plasmid[2] was linearized by *Sal*I and subsequently transformed into *P. pastoris* MF1. The transformants were screened on YND plates without histidine and then on YNM plates. The Mut^+ strains were selected and named as *P. pastoris* MF1-GFP. Similarly, a strain which expressing GFP in Δ*mig1*Δ*mig2* was constructed and named as Δ*mig1*Δ*mig2*-Mit1-GFP. Then the insulin precursor (IP) plasmid pPIC9K/IP, which was constructed previously[5], was linearized by *Pme*I and transformed into *P. pastoris* MF1. The Mut^+ transformants which could grow on the maximum concentration of 0.5, 1, 1.5, 2, 3, 4, 5 and 6 mg/mL G418 respectively were screened. Strains with the maximum production of IP in 500-mL baffled shake flask induced by glycerol were selected and designated as *P. pastoris* MF1-IP.

References

1. Zhang, P. *et al.* Catabolite repression of Aox in *Pichia pastoris* is dependent on hexose transporter PpHxt1 and pexophagy. *Appl. Environ. Microbiol.* **76,** 6108-6118 (2010)
2. Xuan, Y. *et al.* An upstream activation sequence controls the expression of *AOX1* gene in *Pichia pastoris*. *FEMS Yeast Res.* **9,** 1271-1282 (2009)
3. Wang, Y. *et al.* Targeting expression of the catalytic domain of the kinase insert domain receptor (KDR) in the peroxisomes of *Pichia pastoris*. *FEMS Yeast Res.* **9,** 732-741 (2009)
4. Cregg, J. M. *Methods in Molecular Biology*. Vol. 389. *Pichia Protocols*. 2nd ed. (Totowa NJ:Humana Press 2007)
5. Xie, T., Liu, Q., Xie, F., Liu, H. & Zhang, Y. Secretory expression of insulin precursor in *Pichia pastoris* and simple procedure for producing recombinant human insulin. *Prep. Biochem. Biotechnol.* **38,** 308-317 (2008)

Primer	Sequence[a]
**MI1U1**	5-CCGGAATTCGCCGACCTTATTCTTGGAAG-3
**MI1U2**	5-CGCGGATCCGATAAAAAAAATTTTATGGT-3
**MI1D1**	5-ACGCGTCGACCTACTCCTCCGACAATGTTG-3
**MI1D2**	5-ACATGCATGCTTCGGTCACTGATGGGTTGG-3
**Zeo1**	5-CGCGGATCCCCCACACACCATAGCT-3
**Zeo2**	5-ACGCGTCGACTAGTCCTGTCGGGTTT-3
**MI2U1**	5-CCGGAATTCAGCATGTCAAATGTGAACGT-3
**MI2U2**	5-CGCGGATCCTGCGAAGTATCTATTGGTGA-3
**MI2D1**	5-ACGCGTCGACTTGTGACATATAAGTTTGTT-3
**MI2D2**	5-ACATGCATGCGCAGTTGCCCGTAATCAGAC-3
**Kan1**	5-CGCGGATCCAAGGAGATGGCGCCCAAC-3
**Kan2**	5-ACGCGTCGACACTTGAAGTCGGACAGT-3
**NRG15F**	5-CGAGCTCCTGTGCCTATTACCCCCCTT-3
**NRG15R**	5-TCCCCCGGGAACAGATAACCAAAACGGACG-3
**NRG13F**	5-GCTCTAGAGTATTTATTTACGGATTGGA-3
**NRG13R**	5-ACATGCATGCTTCGGCTCCTCTTTCCCA-3
**Hyg1**	5-TCCCCCGGGAGCTTGCCTTGTCCCCGCCG-3
**Hyg2**	5-GCTCTAGATCGACACTGGATGGCGGCGT-3
**MIG1F**	5-AAGGCGGCCGCTACCACTGCCTATCC-3
**MIG1R**	5-ATGGTCGACTTATTTCTTTTCTTGATTTTC-3
**MIG2F**	5-AAAGCGGCCGCTCTACTACTGCTCCCC-3
**MIG2R**	5-GGGGTCGACTTAAGAGTCCGAGTTCATG-3
**GFP1**	5’-TATTTCGAACCATGGGATCTAAAGGTGAAGAATTATTC-3’
**GFP2**	5’-AGAGCGGCCGCCACCTTTGTACAATTCATC-3’
**EcoRI-MIT1-5**	5-CCGGAATTCACCATGGGTAGTACCGCAGCCCCAAT-3
**MIT1-3-XhoI**	5-CCGCTCGAGCTATTCTTCAACATTCCAGTA-3
**SfuI-PRM1-5**	5-CAACTATTTCGAAACCATGGGTCCTCCTAAACATCGGCTG-3
**PRM1-3-XhoI**	5-CCGCTCGAGTTAACTGTCAAAATTTATTGTATCT-3

Table S1. Primers used in this study. [a] Relevant restriction sites are underlined.

Plasmid	Characteristic(s)	Source or reference
**pUC18**	Ampicillin^R; *E. coli* subcloning vector	Invitrogen
**pUC18-MIG1-del**	pUC18 derivative carrying the Pp*MIG1* deletion cassette	This study
**pUC18-MIG2-del**	pUC18 derivative carrying the Pp*MIG2* deletion cassette	This study
**pPIC3.5K**	Ampicillin^R G418^R; P_{AOX1}-based expression vector	Invitrogen
**pPICZ A**	Zeocin^R; P_{AOX1}-based expression vector	Invitrogen
**pRDM054**	Ampicillin^R bleomycin^R hygromycin^R	2
**pGAPZα A**	Zeocin^R; P_{GAP}-based expression vector	Invitrogen
**pGLM1**	pGAPZα A derivative carrying the *GFP-MIG1* fusion gene	This study
**pGLM2**	pGAPZα A derivative carrying the *GFP-MIG2* fusion gene	This study
**pGGM1**	pGAPZA derivative carrying P_{GAP}-Mit1 expression cassette	This study
**pPIC6A**	Blasticidin^R; P_{AOX1}-based expression vector	Invitrogen
**pP6GM1**	pPIC6A derivative carrying P_{GAP}-Mit1 expression cassette	This study
**pGGP1**	pGAPZA derivative carrying P_{GAP}-Prm1 expression cassette	This study
**pP-GFP**	pPIC3.5K derivative carrying P_{AOX1}-GFP expression cassette	This study
**pPIC9K/IP**	pPIC9K derivative carrying P_{AOX1}-(insulin precursor) expression cassette	This study

Table S2. Plasmids used in this study. ^{R}, resistance to indicated antimicrobial agent.

Primer	Sequence
RT-ACT1F	5-CTCCAATGAACCCAAAGTCCAAC-3
RT-ACT1R	5-GACAAAACGGCCTGAATAGAAAC-3
RT-MIT1F	5-GACTAATGACGATGAACTAAG-3
RT-MIT1R	5-TGCTGTTGTTGGTAGAAT-3
RT-PRM1F	5-CGAACTTGATGATGAGAACA-3
RT-PRM1R	5-CATTGGCTATTCCTGAACTG-3
RT-MXR1F	5-ATGCTGCTGATGCTATGA-3
RT-MXR1R	5-GCGGTCTGAATCGTTATTAC-3

Table S3. Sequence of gene-specific PCR primers used in the study.

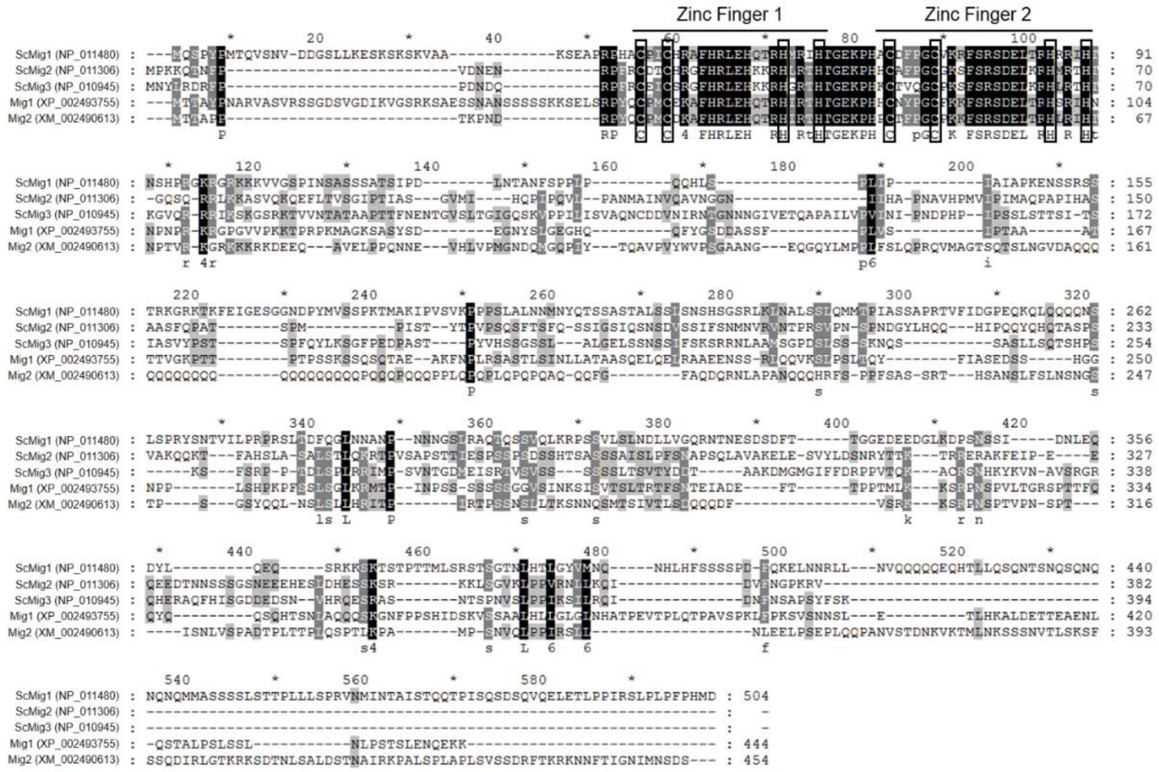

Figure S1. Alignment of Mig proteins in *Saccharomyces cerevisiae* and *P. pastoris*.

Original gels/blots for Figure 1C

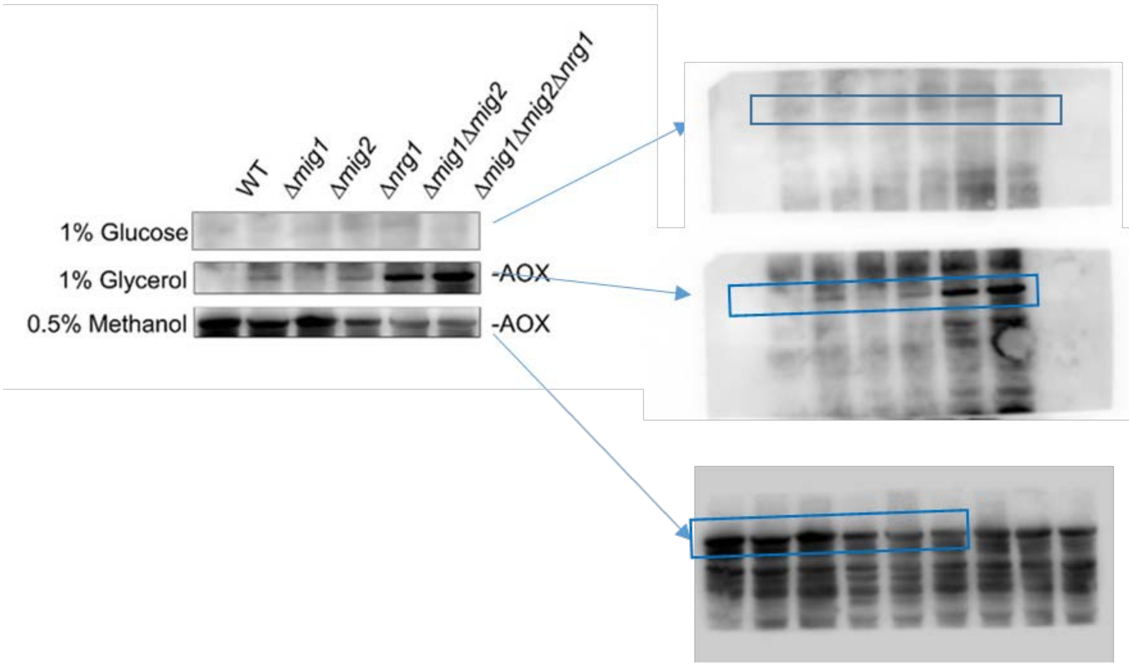

Original gels/blots for Figure 2C

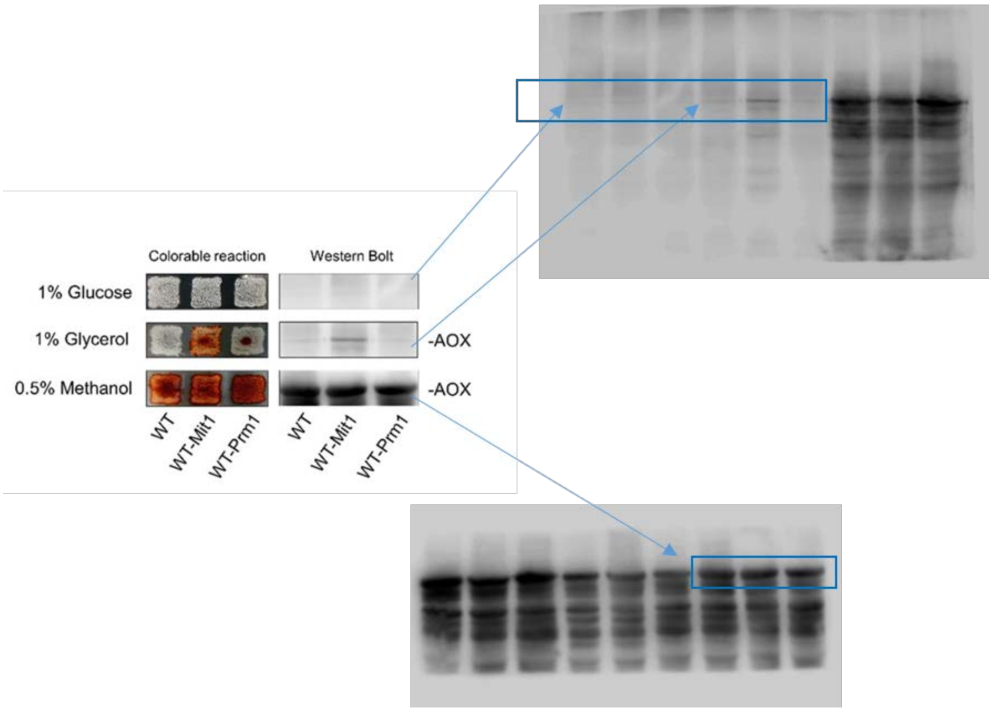

Figure S2. Original gels and blots for figure results (Figure 1C and Figure 2B) shown in the manuscript.

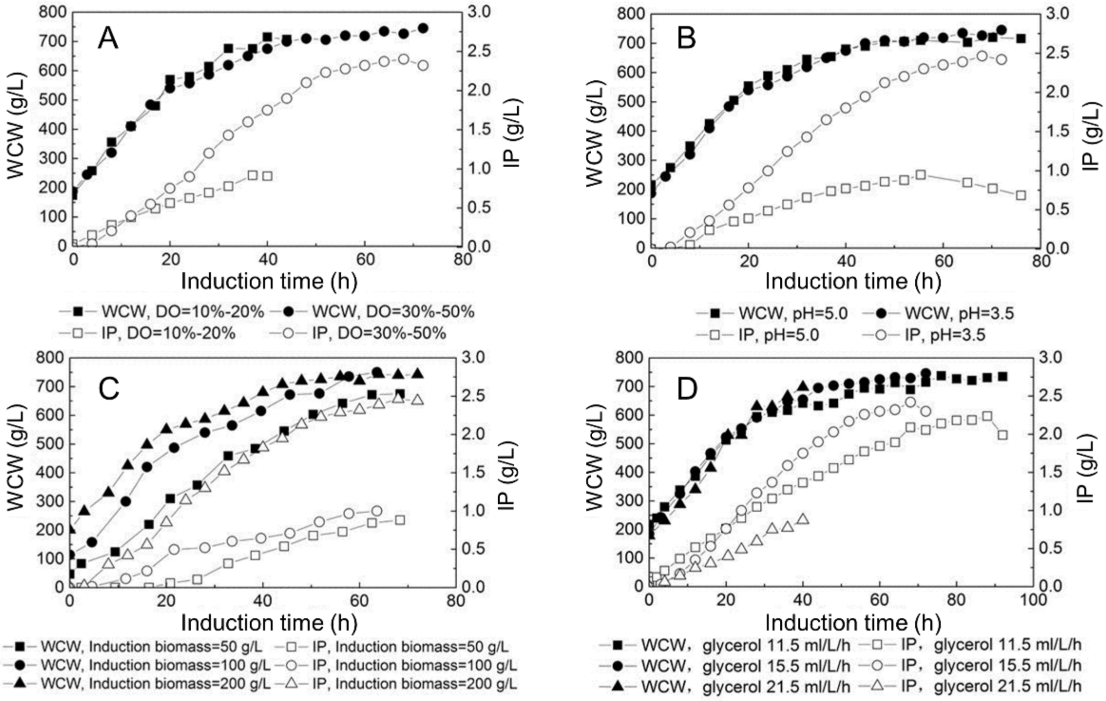

Figure S3. **Effects of DO, pH, induction biomass and induction feeding rate of glycerol on cell growth and insulin precursor expression of the MF1-IP strain.** (**A**) DO; (**B**) pH; (**C**) Induction biomass; (**D**) Glycerol feeding rate.
